# Qualitative study of socio-cultural challenges in the nursing profession in Pakistan

**DOI:** 10.1186/s12912-020-00417-x

**Published:** 2020-04-10

**Authors:** Sidra Abbas, Rubeena Zakar, Florian Fischer

**Affiliations:** 1 Department of Gender Studies, University of the Punjab, Lahore, Pakistan; 2 Department of Public Health, University of the Punjab, Lahore, Pakistan; 3 Department of Population Medicine and Health Services Research, School of Public Health, Bielefeld University, Bielefeld, Germany; 4 Institute of Gerontological Health Services and Nursing Research, Ravensburg-Weingarten University of Applied Sciences, Weingarten, Germany

**Keywords:** Nurse, Nursing, Social challenge, Cultural challenge, Gender-segregation

## Abstract

**Background:**

In a patriarchal social system, a women-dominated profession like nursing is mostly seen as a disempowered group due to its stereotypical image and negative connotations. The low social prestige of this profession is based on the roles typically assigned to men and women to maintain gender identity according to their performance and embodiment. The aim of this study was to explore the social and cultural challenges faced by nurses while creating their professional image within the regional context of Lahore (Punjab) in Pakistan.

**Methods:**

A qualitative research design was chosen to conduct one-to-one, in-depth interviews with twelve nurses. Recruitment was based on purposive sampling from three large public hospitals in Lahore to learn about nurses’ perceptions of social and cultural challenges in the nursing profession. A thematic analysis was conducted using the data analysis software package NVivo 12 Plus.

**Results:**

Cultural values give preference for female nurses. We have identified four major themes related to the social and cultural challenges facing the nursing profession: 1) gender-segregated profession, 2) inappropriate portrayals by the media, 3) issues around marriage settlement, and 4) identity from a religious perspective. These conflicts are affecting the professional status and changing perceptions of nurses, who either do not choose to remain in the nursing profession or do not recommend nursing as a career option. These ongoing constraints are still perpetuating and increasing shortage of nurses within the Pakistani healthcare system.

**Conclusion:**

The present study solely highlights nurses’ perspectives on redefining gender roles and gender integration within the nursing profession. It argues that there is a need for positive portrayals in the media for the removal of public misperceptions related to nursing. This would reduce the shortage of nurses along with increasing retention and improving the quality of healthcare delivered to the public.

## Background

The nursing workforce has faced a variety of socio-cultural challenges which have substantially changed its status [1, 2]. Florence Nightingale, mother of the nursing profession, focused on working in a traditional caring practice, calling womanhood for maintaining harmony in prescribed societal moral values [3]. However, thousands of minority women who played the role like doing more with less, have downplayed the image of the nursing to sisterhood only [4]. Socio-cultural boundaries have affected the nursing profession, as it has been described in studies from Iran [5] and Spain, where also the strong influence of gender roles has been identified [6]. The media continues to portray stereotypical images of nurses being angels of mercy or sexy nurses [7–9]. Hindus believe that female nurses will not receive marriage proposals because they perform night duties and handle male patients [10].

In Pakistan, the inferior status of the nursing profession is related to a variety of stigmas because of its negative portrayal by the media [11], interacting with and touching male patients while caring [12], gender roles distribution on identity basis, and crossing social boundaries in the spotlight of *Purdha* [13], the religious and social practice of female seclusion among Muslim communities. All of these stigmas are intensifying the decrease in nurse-to-patient ratio [14, 15].

In this study, the theory of gender performativity developed by Butler [16] and Le Blanc [17] was applied, along with the concept of objectification, in order to highlight this performativity and uncover its linkage to the situated identity of nurses within the given social context. This theory emphasizes the constructed identity of nurses which they have gained through their performativity of specific behaviours by following the virtue script of femininity. This results in a closure of culturally conditioned norms [17] and causes gender inequality for male nurses. The stereotypical portrayal and objectification of nursing [18] has placed nurses in a subservient position to physicians and compelled them to give their emotional and physical labour to others, which has devalued the image of the nursing profession. The social and moral dilemma of receiving unflattering descriptions [19] are reasons for a possible loss of self-belief, which affects the delivery of high-quality healthcare.

The present study aims to explore the social and cultural challenges affecting the image of the nursing profession, changing nurses’ perceptions and causing a shortage of nurses in clinical practice within the context of Lahore (Punjab), Pakistan. This study is significant, because it identifies the current status of nursing profession solely from nurses’ perspectives about their clinical experiences. The positive depiction of nursing profession to general public through media will remove socio-cultural stigmas of stereotypical image. Gender integration in nursing would increase acceptance of male nurses within nursing profession. In addition, it will ultimately reduce gender gaps in this profession. By redefining gender roles in caring practice from motherhood to an innovative profession, it would improve quality of healthcare and remove disparities of its public image. Such issues are very relevant and demand highlighting the problems of recruitment and retention in nursing profession.

## Methods

A qualitative research approach was applied. Twelve one-to-one, in-depth interviews – each of them lasting about 90 min – were conducted until saturation point was reached. The interviews were based on a semi-structured interview guide, which was developed for this study (Additional file 1). The foremost questions of this study were: What kind of social challenges shape the image of nursing profession? What are the cultural issues for nursing profession which are affecting its image? What are the perceptions that prevail for nurses and nursing profession from a religious perspective? What type of gender differences and traditional values exist altering the status of the nursing profession? How is media portraying the image of the nursing profession? Furthermore, the prompt and subsidiary interrogation was part of the interview guide in order to clarify responses.

### Participant recruitment

Three government hospitals were selected for the recruitment of study participants, because they are large public and teaching hospitals for nursing students and professionals in Lahore. Recruitment was based on purposive sampling. Study participants were recruited after we approached the senior authorities, including members of the research board and medical and nursing superintendents. Study participants included registered staff nurses, head nurses and deputy chief nursing superintendents from the emergency department, intensive care unit, and administration. Nurses of different ranks were considered suitable for interviewing due to their practical experience and sound knowledge of the supervision and training of nurses, and their continuous day-to-day interactions with clients [20]. All the nursing professionals had studied and worked most of the time in Pakistan (Table 1). Interviews were conducted within an eight-week period between September and November 2018.

**Table 1:** Demographic profile of nursing professionals

Nurses	Qualification	Number of participants	Average years of experience
Staff nurses	Diplomas	4	5 years
Head nurses	Diplomas + specialty	4	10 years
Deputy chief nursing superintendent	Diplomas + post-graduation	4	18 years

### Data analysis

The interviews were recorded and translated verbatim into the English language [21]. Data analysis was conducted using the software package NVivo 12 Plus. We used a thematic analysis, in which transcribed data was added to form common codes by combing point of commonality. Data analysis went through the stages of compiling 1) nodes, 2) leading nodes, and then 3) common themes. Table 2 explains the process of data analysis. We used an iterative process, in which similar nodes and leading nodes were organised after the interviews with the first three participants. Further interviews with participants four to ten consolidated the same nodes. At this point, no new information was forthcoming. The last two interviews were organised to make sure the point of saturation about common themes had been reached.

**Table 2:** Nodes, leading nodes, and common themes of socio-cultural challenges

Nodes	Leading nodes	Common themes
Assigned roles of men and women within society, for women motherhood and man bread earner	Gender roles on basis of gender identity	Segregated identity as a male and female nurse
Nurses are hard, dirty and rude	Silent voices	Negative image and inappropriate portrayal in media
Performing night duties outside home with male colleagues	Not reputable profession	Issues around marriage settlement
Religious families want to attend their clients form preferably female nurses	Cultural stigmas	Identity from religious perspective

## Results

The present study describes the nurses’ point of view about the social and cultural challenges that they may face while creating their professional image. Traditional historical aspects of nursing indicate that the occupation embodies universal characteristics of the feminine. The results of this study revealed four key themes that emerged from the thematic analysis, which are interlinked with themes and sub-themes [22] relating to the social and cultural challenges facing the nursing profession: 1) gender-segregated profession, 2) inappropriate portrayals by the media, 3) issues around marriage settlement, and 4) identity from a religious perspective (Table 3).

**Table 3:** Themes and sub-themes of social and cultural challenges in nursing

Themes	Sub-themes
1. Gender-segregated profession	•Less acceptance or opportunities for male nurses •Only feminine profession
2. Inappropriate portrayal in media	•Low profile professional as subordinate to doctors •Portraying in caring role but not skillful professionals
3. Issues around marriage settlement	•Rejection, leave job or hide identity for marriage proposal •Second class citizen belongs to less educated families
4. Identity from religious perspective	•Touching male clients in caring work makes nurses impure •Concept of seclusion of women from the sight of male

### Gender-segregated profession

The first theme that emerged from the study findings was segregation of nursing professionals on basis of their gender identity. Muslim societies mainly give preference to female nurses to care for their patients [23]. Butler [24] argued that the concept of a “natural sex” predates socialisation, which is the basis for being gendered. In patriarchal societies, it is obligatory for women to fulfil the needs of men [25], which perpetuates strict adherence to gendered roles. If men shuffle their role performance with female roles, their gender-identity gets lost with their pre-defined notions of manhood [26]. A staff nurse summarized this aspect as follows:

*My female friend’s brother does not consider nurses to be good professionals – neither male nor female nurses. His point of view about nursing professionals is no more positive. I wish he would marry a nurse so he could understand their situations and how the judgement of societies is purely based on cultural stigmas.* (Participant 8)

### Inappropriate portrayals in the media

The study results illuminate another important theme related to the visibility and portrayals of the nursing profession in the media. The media disseminates a stereotypical image of nurses, along with the concept of objectification, which involves ignoring a person’s intellectual capacities only because of her gender identity. The public objectifies female nurses as handmaidens of doctors and to provide pleasure for their sexualized gaze. Buresh, Gordon and Bell [27] explained in their book “From Silence to Voice: the Truth about Nursing” that a key cause of the global nursing shortage is the lack of real respect for nurses, which devalues the nursing profession on the basis on performativity [24]. A ward head nurse from the intensive care unit, with 12 years of work experience, said:

*I remember a drama serial called “Nurse” that was aired during my childhood. The image of nurses was that they took money from patients’ relatives for the sake of their poor families. The story of this drama was about kidnapping a newborn by a nurse for the sake of money.* (Participant 5)

### Issues around marriage settlement

Marriage settlements for nurses in this society also emerged as a challenge. The intimate nature of the nursing profession violates the social and cultural taboos in relation to bodily contact [28]. The public uses derogatory slang for nurses, such as calling them “sleeping pills” for physicians. Butler [29] explained the implications of language, which can be manipulated and used as a source of power in patriarchal culture. In this way, speech acts on women’s bodies as on a passive medium, in such a way that, in nursing, public slang leads to a sense of unsatisfactory performance. A head nurse with 12 years of work experience commented:

*My former partner broke off our engagement just because I wanted to continue within the nursing profession. Now my present partner does not want me to continue further with my job in nursing. He wants me to leave this profession and join another. And just to satisfy him, I am studying a Master of Science in Psychology besides my nursing job.* (Participant 9)

### Identity from a religious perspective

The final theme emerging from the study results was the social identity of nurses from a religious perspective. Muslim societies stigmatise nurses for their interactions with male patients [12]. Within the nursing profession, the natural touching of bodies and patient privacy is an integral part of the nurse–patient interaction, regardless of culture. Butler [19] explained that gendering is a way to mark persons as subjects deserving of respect. Something strange happens to women, turning them into objects because of their feminine identity. In Catholicism and Islam, the covering of the female head signifies humility, submissiveness and dutifulness, and the nurse’s uniform also shapes a negative stereotypical image of their gendered, grounded nature within a dominant organisational culture [30]. A deputy chief nursing superintendent shared her experience of 18 years in the following way:

*I had the experience of attending a male patient who belongs to a religious family. His wife said to me: “I am very much worried about Pakistani nurses; how will you protect yourself on the day of Akhrat (The Day of Judgment)?”* (Participant 10)

## Discussion

The current study aimed to explore nurses’ perceptions about challenges that they might have faced during their clinical experience as they progress from being a student nurse to professional nurse. This has revealed very thought-provoking findings and points to the needs to discuss the situated identity of nursing [31]. These experiences explain why increasing numbers of nurses still hold the intention to leave their jobs, which is causing severe skills shortages in Pakistan.

Socio-cultural norms have reinforced the concept of gender-based segregation on the basis of role assignment. This has caused less acceptance of male nurses, because society does not consider nursing a suitable profession for men. The inherent qualities, traditional household roles, and fundamental nature of work are preferable characteristics that adhere to female nurses only. The gendered context is the construction of an identity of a person which is highly relevant to the division of labour work on behalf of performativity. In patriarchal societies, the continuous observance of gender roles denies the entry of men into female-dominated professions, but when men do come to join the nursing profession, they cannot sustain their prevailing role of hegemonic masculinity [26].

The presence of male nurses in the healthcare system changed over time. On the one hand, male nursing is beneficial for sharing the burden of duties, workload and gender balance within the profession. But on the other hand, male nurses are seen as separate identities in this feminine domain, which results in feelings of isolation and affirms their sense of not belonging or being acknowledged as part of the profession [32]. Gul [11] explained that the gender of nurses and the nature of nurses’ work both affect the image of the nursing profession and depict a scene of domestic doing that relates to femininity only. Professional identity may be highly relevant to female nurses and occupational career assessment may be highly relevant to male nurses [33]. If a man chooses nursing as a profession, then his motive will be to earn money as the breadwinner of his family. Boughn [34] explained that male nurses tend to place more importance on salary.

Media portrayals are not accurate about physical appearance, dress codes or doctor–nurse relationships. This is shown in the very stereotypical media portrayal of low-profile professionals. The on-screen representations of female nurses are characterised by their overestimation of the profession’s foundations, whereas male nurses are presented very rarely in the nursing profession [35]. An examination of 36,000 feature films highlighted that, in these films, nurses were depicted as self-sacrificing, strong and confident professionals in early times. The most popular medical dramas and television shows, like ER and Grey’s Anatomy [27], offered audiences the chance to learn very little about nurses, because physicians were portrayed as the dominant and appreciated health professionals in the medical field, whereas nurses were defined to play their subservient roles.

Studies explained that the nursing profession has partly influenced their invisibility in media by themselves for not raising their silent voices. The mass media image of nursing transformed its professional status from cliché to provocative vocation of female which is continuously damaging the public perceptions. For that reason, nursing professionals do not understand their position with respect to their profession and do not recommend others to join nursing as a career. Their reshaped conflicted identity receives less recognition of more powerful roles, because they have not been considered as autonomous healthcare providers or advocates of the clients. Rather, they are only seen as care workers within patriarchal organizational systems who are just working as competent machines of dominant physician [36, 37].

In this study, most of the nursing professionals shared stories about facing issues around marriage settlement, because of the nature of nurses’ work and working environment. Nurses usually are not preferable choices for men in terms of marriage and face difficulties in finding life partners. This is due to their night shifts in hospitals as well as negative perceptions about nurse-doctor interactions and gender mixing at the workplace. Nurses’ work has been described in negative terms such as hard, dirty, and with minimal chances of marriage and family, as well as low paid [38]. Nurses who want to continue with their job after marriage are usually mounted by their husbands to discontinue their job. This results in less satisfaction of work performance in delivering healthcare.

Society does not give due regard to nurses and they have to face rejection in marriage proposals. Sometimes marriage settlement agreements include the conditions that they must either leave the nursing profession or not be accepted for marriage [39]. Hiding their professional nursing identity is one of the cultural practices employed by nurses to save the honour of their family and to receive social support. Nurses do not often recommend others to join their profession, unless they do so for the salary and get remuneration for their work as economic support.

The current study also collected information about nurses’ perspectives on religious aspects which is a cornerstone of this profession and influences nurses’ attitudes during nurse-client human interaction. Religious considerations are important in terms of the process of caring for and touching male patients during clinical practice. In Western societies, religion does not always have much influence upon people’s attitudes as it does from an Islamic perspective. The learned touching behaviour is the foundation of nursing, but this can be significantly changed by the nurses’ socio-cultural background and practice in nursing school [40].

Physical touch in nursing brings humanity and compassion in work patterns of care-oriented tasks. It provides emotional containment and empowers clients’ wellness. Nurse’s try to reduce caring tasks on their male clients to reduce the time of physical touching, although handling patients’ bodies has been labelled as the essence of the nurses’ role [41], but in Pakistani culture many dilemmas exist in society because people consider nurses to be impure from a religious point of view as they have to touch male patients and perform night duties outside the home. This crosses cultural boundaries of mobility and modesty and becomes a confusing puzzle for social fabrication of society [42].

### Limitations

The overall limitations linked with qualitative research also apply to the interpretation of this study. One might expect that the study population is quite selective, as respondents have been recruited from large public and teaching hospitals. Furthermore, their willingness to participate in the study may lead to a selection bias. For that reason, it is not clear in how far the results are generalizable. A further limitation may occur in the form of data analysis. Although a thematic analysis is based on the articulated phrases, not expressed attitudes and non-verbal information has not been included. Religious practices and beliefs play a major role in perceptions and practices in the study region. Therefore, these aspects may have influenced the perceptions and reports of the study participants.

## Conclusion

The current research has provided relevant insights into the interviewed nurses’ perceptions about the challenges they face due to gender discrimination, negative portrayals in the media, marital issues, and religious identity. These challenges, which the nurses had faced throughout their careers, from being a student nurse to their time as nursing professionals, caused inequalities for male nurses. For that reason, it has become a stigma which prohibits men from joining the nursing profession. Improper representation in the media of nurses being low-profile professionals, handmaidens to doctors, and tools of emotional gratification for the public has had negative implications for the image of the nursing profession. Adopting behaviour of having both overt and covert identities in order to meet the set standards of society for marriage settlements, to quit nursing or to not perform night duties on male patients ultimately means losing one’s self-identity as a professional. To overcome these challenges, there is a need to focus on adopting professionalism and boosting the silent voices of nurses through the media. The results may have implications on the nursing practice itself, but also on nursing education in particular, as more men are willing to be employed in the nursing profession.

## Supplementary information

**Additional file 1.** Interview guide.

## Data Availability

Pseudonymised transcripts are available from the corresponding author upon reasonable request.
