# Supplementary material for: Qualitative study of socio-cultural challenges in the nursing profession in Pakistan
Source: BMC Nurs. 2020 Apr 10;19:20. doi: 10.1186/s12912-020-00417-x (PMC7147052; doi:10.1186/s12912-020-00417-x)
Supplement: Supplementary file 1 — Additional file 1. Interview guide. [file 12912_2020_417_MOESM1_ESM.pdf]

Interview guide

Qualitative Study of Socio-Cultural Challenges in the Nursing Profession in Pakistan

Question	Prompts	Probing questions
What kind of social challenges shape the image of the nursing profession?	What are the public perceptions about nurses?	• How much respect do you receive from society as a nursing professional?<br>• Do people behave according to societal image of nursing?<br>• In how far do you understand that the main duty of your job is to nurture your clients?
What are the cultural issues for nursing profession which are affecting its image?	How do people respond to your professional identity?	• In how far are you facing any marital issues?<br>• Have your in-laws suggested you to leave your job for family honor?<br>• What are your perceptions about assigned labels to nursing profession?<br>• What kind of language does the public use for nurses? Can you tell some words in any language?
What are the perceptions that prevail for nurses and nursing profession from a religious perspective?	What does the public think about your work patterns of care?	• What is the public image of nurses?<br>• How is the image related to their night duties?<br>• Does the public consider you impure because of touching male patients?<br>• Do you have the intention to quit your job in such scenarios?

		• How much do you recommend others to join the nursing profession?
What type of gender differences and traditional values exist altering the status of the nursing profession?	In how far do you think nursing is a gender-neutral profession?	• What are the implications of gender roles?<br>• In how far do gender differences discriminate your identity being a nursing professional?<br>• Is gender-segregation benefiting female nurses?<br>• Do you have to follow virtue scripts in your role as a female nurse?<br>• Do you support male nursing?<br>• How do you judge society's acceptance of male nurses?
How is media portraying the image of the nursing profession?	In how far are nurses visible in media?	• Is media depicting the stereotypical image of nursing?<br>• How do stereotypical roles presented in media discredit the social reputation of nurses?<br>• What kind of programs on television are related to nursing?<br>• In how far are nurses interested to raise their voices?<br>• In how far are nurses portrayed subordinate to doctors?
